# Dynamic changes in CD44v-positive cells after preoperative anti-HER2 therapy and its correlation with pathologic complete response in HER2-positive breast cancer

**DOI:** 10.18632/oncotarget.23914

**Published:** 2018-01-04

**Authors:** Teruo Yamauchi, Jose Rodrigo Espinosa Fernandez, Chiyo K. Imamura, Hideko Yamauchi, Hiromitsu Jinno, Maiko Takahashi, Yuko Kitagawa, Seigo Nakamura, Bora Lim, Savitri Krishnamurthy, James M. Reuben, Diane Liu, Debasish Tripathy, Helen Chen, Naoko Takebe, Hideyuki Saya, Naoto T. Ueno

**Affiliations:** ^1^ Division of Medical Oncology, St. Luke's International Hospital, Tokyo, Japan; ^2^ Department of Breast Medical Oncology, The University of Texas MD Anderson Cancer Center, Houston, TX, USA; ^3^ Department of Clinical Pharmacokinetics and Pharmacodynamics, Keio University School of Medicine, Tokyo, Japan; ^4^ Department of Breast Surgery, St. Luke's International Hospital, Tokyo, Japan; ^5^ Department of Surgery, Teikyo University School of Medicine, Tokyo, Japan; ^6^ Department of Surgery, Keio University School of Medicine, Tokyo, Japan; ^7^ Department of Breast Surgical Oncology, Showa University School of Medicine, Tokyo, Japan; ^8^ Department of Pathology, The University of Texas MD Anderson Cancer Center, Houston, TX, USA; ^9^ Department of Hematopathology Research, The University of Texas MD Anderson Cancer Center, Houston, TX, USA; ^10^ Department of Biostatistics, The University of Texas MD Anderson Cancer Center, Houston, TX, USA; ^11^ Cancer Therapy Evaluation Program, National Cancer Institute, Rockville, MD, USA; ^12^ Division of Gene Regulation, Institute for Advanced Medical Research, Keio University School of Medicine, Tokyo, Japan

**Keywords:** CD44v, predictive, biomarker, HER2, breast cancer

## Abstract

Chemotherapy has been reported to increase the proportion of cancer stem cells (CSCs) and to promote epithelial-mesenchymal transition (EMT) phenotype changes. Anti-HER2 therapy may provide a strategy for eliminating CSC and EMT, which contribute to therapeutic resistance. No study has determined the changes in the quantity or characteristics of CSCs or circulating tumor cells (CTCs) with EMT phenotype during preoperative anti-HER2 therapy, and whether these changes correlate to response to dual anti-HER2 therapy. In a prospective clinical trial to evaluate pharmacodynamic biomarkers, 18 patients with operable primary HER2-positive breast cancer received dual anti-Her2 preoperative therapy with trastuzumab and lapatinib with paclitaxel. Proportions of tumor cells with CSC characteristics and EMT markers in CTC's were estimated at baseline, after 6 and 18 weeks of preoperative therapy to determine the quantitative cutoff value to predict pathologic complete response (pCR). Out of 18 patients, 8 (44%) had a pCR; 5 of these 8 patients (62%) were positive for CD44v at baseline and none were positive on the 6-week biopsy. In contrast, 6 of the 10 patients without pCR exhibited persistent levels, or enrichment of CD44v proportion and expression at 6 and 18 weeks (p=0.0128). Other biomarkers were not statistically significant predictors of pCR. Enrichment of CD44v-positive tumor cells after dual anti-HER2 therapy alone may predict poor response to dual anti-HER2 therapy plus chemotherapy.

## INTRODUCTION

Preoperative chemotherapy has become an accepted approach for operable breast cancer since it provides the option for breast-conserving surgery in women presenting with large tumors, allows drug resistance to be identified early, and gives an opportunity for physicians to monitor the tumor's sensitivity to specific therapies [1]. The addition of trastuzumab to preoperative chemotherapy for HER2-positive (HER2+) breast cancer has been tested in several schedules and combinations and has led to improved pathologic complete response (pCR) rates, which seem to correlate with improved outcomes and clinical benefit [2, 3] and has become a standard of care for HER2-positive breast cancers. Lapatinib is a reversible EGFR/HER2 tyrosine kinase inhibitor that targets HER2 by different mechanisms than the ones through which trastuzumab targets HER2. The NeoALTTO study compared dual anti-HER2 therapy with lapatinib plus trastuzumab versus single anti-HER2 therapy with lapatinib or trastuzumab as preoperative therapy given concurrently with weekly paclitaxel in women with early HER2+ breast cancer. The study showed that dual anti-HER2 therapy produced a superior pCR rate, which was associated with a statistically significant improvement in event-free survival and overall survival, only in those patients who achieved pCR [4, 5], therefore the development off a new biomarker for predicting response to anti-HER2 therapy is of great interest.

Cancer stem cells (CSCs) are a subpopulation of tumor cells that have a tumorigenic potential much greater than other cancer cells. CSCs have the ability to self-renew and to generate differentiated tumor cells and contribute to the overall organization of a tumor. CSCs are resistant to various therapeutic interventions and, thereby, able to survive and evolve after different kinds of treatments [6]. In breast cancer, CSCs are characterized by CD44 expression with absent or low CD24 expression (CD44+CD24-/low) or by increased aldehyde dehydrogenase 1 (ALDH1) enzymatic activity or expression; cells displaying either of these phenotypes on gene expression profiling have been associated with tumorigenesis, invasion, and poor prognosis [7]. CD44 exists in numerous isoforms generated through alternative mRNA splicing [8]. The variant isoforms (CD44v) are abundant in epithelial-type carcinomas but not expressed in any immune cells and fibroblasts in tumor tissues. CD44v has been found to stabilize the expression of the cysteine transporter xCT on cell membrane and to promote the production of glutathione which reduces reactive oxygen species [9]. CD44v-positive cancer cells are therefore resistant to oxidative stress and possess stem-like properties [10–12]. High enzymatic activity of ALDH1 and its expression determined by IHC are also proposed as a CSCs marker in breast cancer. Breast cancer cells with high enzymatic activity of ALDH1 are responsible for chemo resistance [13].

Another feature that may be an indicator of early tumor relapse and shorter survival in breast cancer patients are circulating tumor cells (CTCs) [14, 15]. Epithelial-mesenchymal transition (EMT) plays a key role in the invasiveness of cancer cells and is relevant to the acquisition of CSC-like characteristics such as high tumor initiating activity and chemoresistance. In breast cancers, CTCs with EMT phenotype are associated with poor prognosis [16, 17].

Conventional chemotherapy for breast cancer destroys non-stem cells while sparing CSCs [18]. In contrast, anti-HER2 therapy may target CSCs, and trastuzumab's activity may be due in part to its ability to inhibit CSCs in HER2+ tumors. HER2 appears to be a key driver of CSCs even in the absence of this receptor's phenotypical overexpression or amplification [19]. Breast biopsy specimens after neoadjuvant chemotherapy have demonstrated increased numbers of CSC biomarkers following therapy [20, 21]. In an analysis of the expression of breast CSC biomarkers and clinical outcomes of patients treated with trastuzumab, CD44+/CD24- was found to be prognostic of poor outcome and predictive factor of response to trastuzumab in patients treated with adjuvant therapy but not those treated for metastatic disease [22]. Li et al [23] reported that the proportion of CD44+/CD24- cells in breast cancer increased during preoperative conventional chemotherapy. However, in a separate group of breast cancer patients with HER2 amplification treated with lapatinib plus conventional chemotherapy, this cell population did not increase. These findings suggest that breast cancer has an intrinsically chemoresistant cellular population, defined as CD44+/CD24-, and that anti-HER2 therapy could eradicate this population of cells or CSCs in HER2+ breast cancer. Anti-HER therapy may benefit patients with tumors that overexpress HER2 in the CSC compartment [24, 25]. The findings reviewed above suggest that the molecular characterization of CSCs and CTCs with EMT features may provide clinicians with surrogate markers for assessing and predicting treatment response in breast cancer patients [26]. There has not been a comprehensive study to determine the changes in the quantity or characteristics of CSCs or circulating tumor cells (CTCs) with EMT phenotype during preoperative anti-HER2 therapy, and whether these changes may predict response to dual anti-HER2 therapy.

A biopsy after a brief exposure to a specific therapeutic strategy (“adaptive response biopsy”), can be used to direct target modulation that is induced by specific therapeutics [27, 28], and may help clinicians determine optimal breast cancer management. The real benefit of adopting the use of adaptive response biopsy to deliver the most optimal preoperative treatment strategy has yet to be assessed, especially in trials where potential predictive biomarkers are being evaluated.

Another potential predictor of therapeutic efficacy, in addition to change in the CSC population and CTCs with EMT features, is the ratio of phosphorylated to non-phosphorylated receptor tyrosine kinases (e.g., HER2, EGFR, VEGFR). In a multi-institutional phase I/II study of erlotinib plus bevacizumab for recurrent or metastatic squamous cell carcinoma of the head and neck, higher ratios of tumor cell pVEGFR2 to total VEGFR2 and higher ratios of endothelial cell pEGFR to total EGFR in pretreatment biopsy samples were associated with complete response and tumor shrinkage [29].

Based on the aforementioned findings, we hypothesized that in patients with HER2+ breast cancer treated with the combination of lapatinib and trastuzumab plus cytotoxic chemotherapy, a decrease in the CSC population correlates with response to therapy. To test this hypothesis, we studied the impact of trastuzumab plus lapatinib delivered to patients with HER2+ breast cancer on i) CSC biomarkers (CD44v and ALDH1) in tumor tissues, ii) EMT markers (TWIST1, SNAIL1, SLUG, ZEB1, and FOXC2) in CTCs in peripheral blood, and iii) the ratios of phosphorylated to phosphorylated EGFR (pEGFR/EGFR), HER2 (pHER2/HER2), ERK (pERK/ERK), and Akt (pAkt/Akt) in tumor tissues. We determined whether changes in the quantitative values of these biomarkers between baseline and predefined points during preoperative systemic therapy predicted pCR or non-pCR.

## RESULTS

### Patient characteristics

Eighteen patients with operable primary HER2-positive breast cancer received dual anti-Her2 preoperative therapy with trastuzumab and lapatinib with paclitaxel. All patients received lapatinib and trastuzumab for 6 weeks, and then completed the following 12 weeks of concurrent anti-HER2 therapy with weekly paclitaxel, and underwent surgery. All 18 patients were assessable for clinical and pathologic response and biomarker assay at the points previously described. Thirteen patients (72%) presented with stage II disease, and 5 patients (28%) presented with stage III disease. Six patients (33%) had HER2+/hormone receptor-positive disease, and 12 (67%) had HER2+/hormone-receptor-negative disease (Table 1).

**Table 1:** Patient characteristics

Characteristic	n(%)			*P*
	Total (N=18)	pCR (N=8)	Non-pCR (N=10)	
T category^*^				
T2	14 (78%)	6(43%)	8(57%)	.2288
T3	2 (11%)	2(100%)	0	
T4b	2 (11%)	0	2(100%)	
N category^*^				
N0	7 (39%)	2(29%)	5(71%)	.7089
N1	8 (44%)	4(50%)	4(50%)	
N2	1 (6%)	1(100%)	0	
N3	2 (11%)	1(50%)	1(50%)	
ER status				
+	12 (67%)	5(42%)	7(58%)	1.000
-	6 (33%)	3(50%)	3(50%)	
PR status				
+	9 (50%)	2(22%)	7(78%)	
-	9 (50%)	6(67%)	3(33%)	

Abbreviations: ER, estrogen receptor; pCR, pathologic complete response; PR, progesterone receptor.

^*^TNM staging system for breast cancer according to the American Joint Committee on Cancer.

### Clinical response

Eight patients (44%) achieved a pCR after dual anti-HER2 therapy followed by concurrent trastuzumab, lapatinib, and paclitaxel (Table 1). Of the 8 patients with a pCR, 5 (63%) had estrogen-receptor-positive and 3 (37%) had estrogen-receptor-negative disease (Table 1).

### Biomarker assays

**Figure 1:**
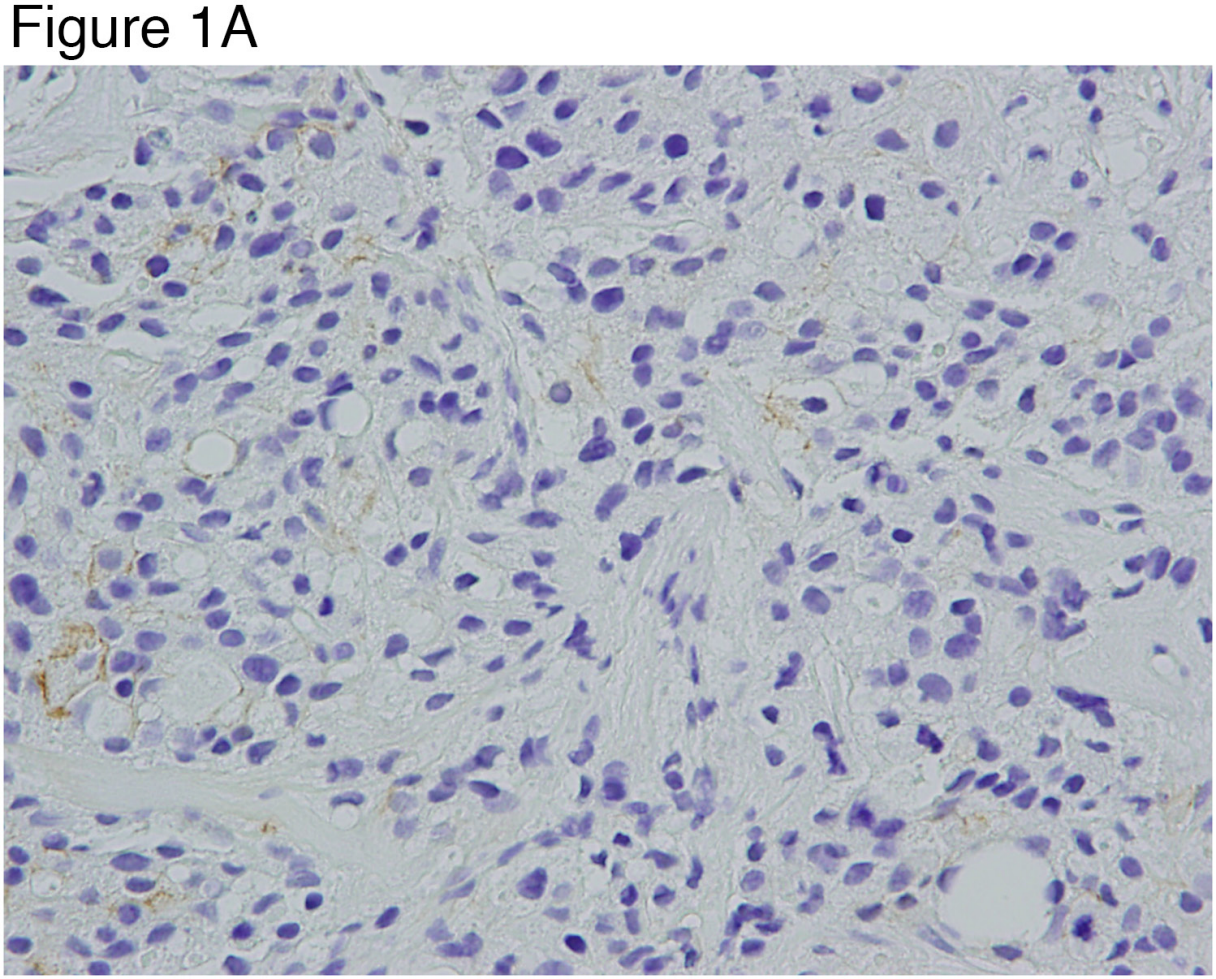

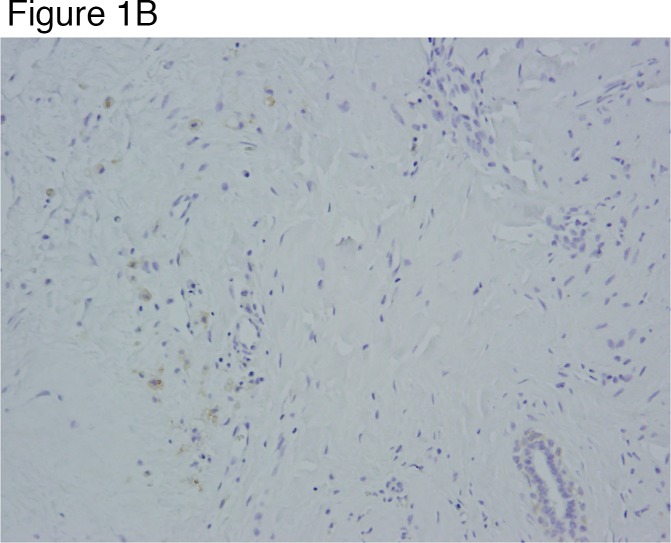
Illustration of a case of invasive ductal carcinoma at baseline as shown in **(Figure 1A)** that was entirely negative for CD44v by immunohistochemical staining (x20). **(Figure 1B)** There is no evidence of residual tumor in the final surgical specimen (pCR). Illustration shows the tumor bed with fibrosis and few scattered inflammatory cells without any evidence of residual tumor (x20).

**Figure 2:**
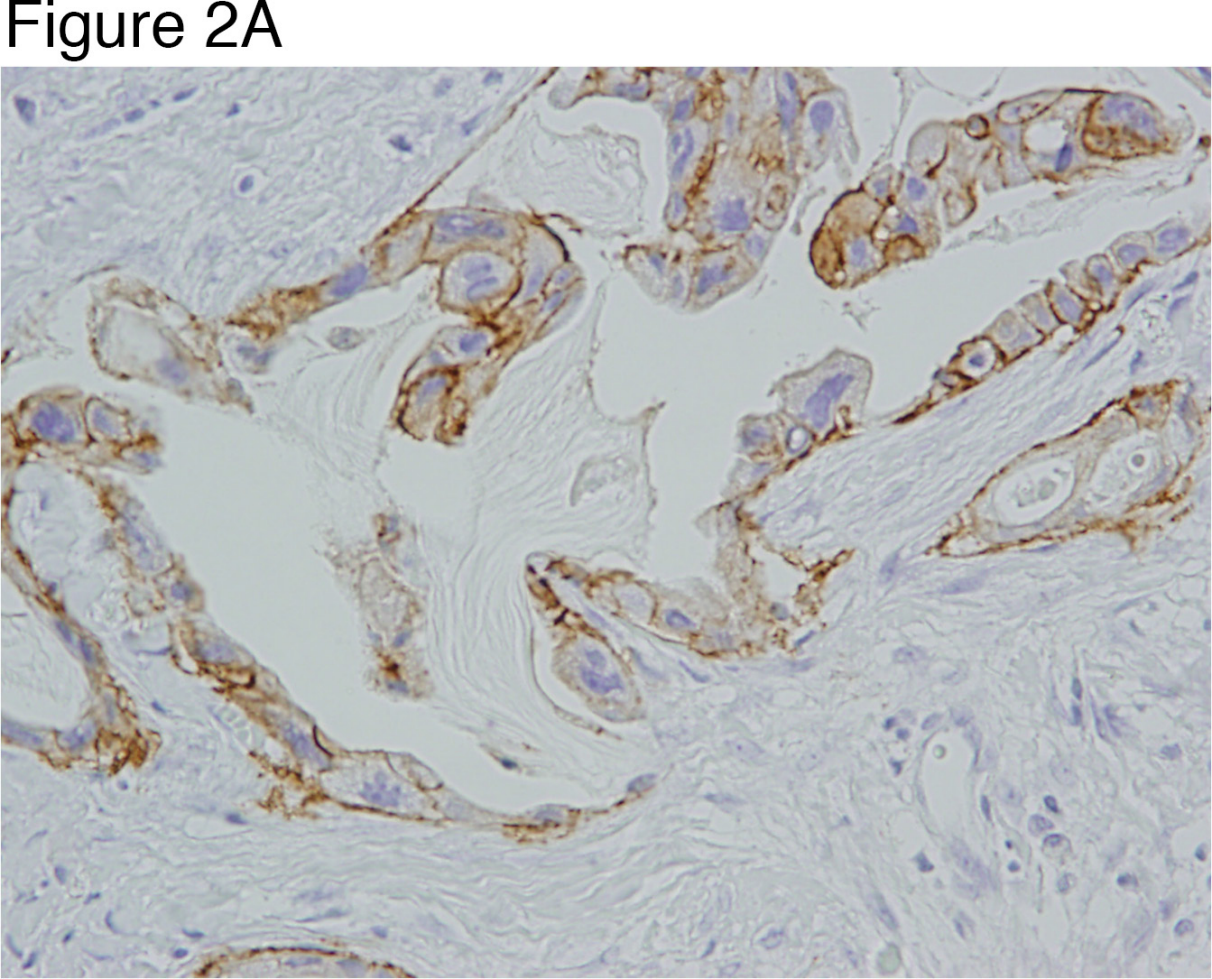

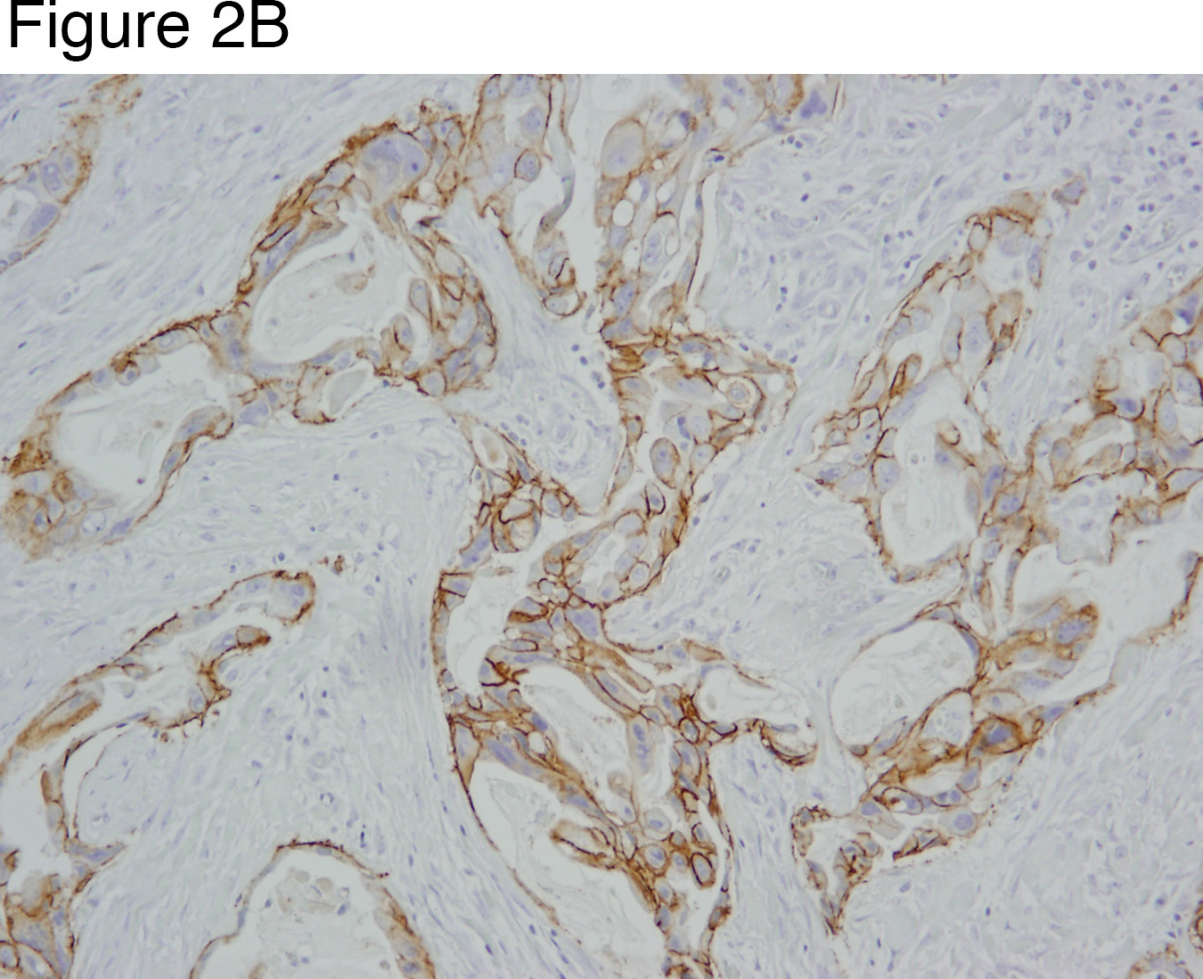
Illustration of a case of invasive ductal carcinoma that shows membranous staining for CD44v in nearly all tumor cells at baseline (x20) **(Figure 2A)**. The residual tumor following neoadjuvant chemotherapy shows persistent positivity for CD44v (x20) **(Figure 2B)**.

ALDH1 expression, EMT markers in CTCs, and ratios of phosphorylated to phosphorylated receptor tyrosine kinases in tumor tissue were not statistically significant predictors of pCR (Supplementary Table 2). The patterns of change in CD44v+ cells in tumor tissue over the treatment course predicted pCR. Of the 8 patients who achieved a pCR, 5 had CD44v+ cells at baseline but none of these 8 patients had CD44v+ cells in the 6-week biopsy specimen. On the other hand, six of the 10 patients (60%) without pCR showed either persistence of CD44v expression or increase in the proportion of CD44v-expressing cells or increase in the intensity of CD44v staining between the baseline biopsy specimen and in the 6 week biopsy specimen, the surgical specimen, or both (p=0.0128, comparing the number of patients without CD44v+ cells using 6-week or surgery biopsies between pCR and non-pCR groups) (Figures 1 Figure 2) (Tables 2 and 3).

**Table 2:** CD44v and pathologic response

Patient	Before Study			End of Week 6			Surgery			Response
	Tumor Cells	% of Cells Positive for CD44v	Intensity of CD44v Staining (0-3+)	Tumor Cells	% of Cells Positive for CD44v	Intensity of CD44v Staining (0-3+)	Tumor Cells	% of Cells Positive for CD44v	Intensity of CD44v Staining (0-3+)	
1	+	0	0	-	0	0	-	0	0	pCR
2	+	0	0	+	0	0	+	0	0	cPR
3	+	0	0	+	20	3+	+	5	2+	cPR
4	+	0	0	-	0	0	-	+	0	pCR
5	+	50	3+	+	10	3+	+	100	3+	cPR
6	+	0	0	+	0	0	+	5	1+	cPR
7	+	30	3+	-	0	0	-	0	0	pCR
8	+	0	0	-	0	0	-	+	0	pCR
9	+	80	3+	+, scant	0	0	+	70	3+	cPR
10	+	10	3+	-	0	0	-	+	0	pCR
11	+	0	0	+	20	3+	+	0	0	cPR
12	+	100	3+	+	100	3+	+	100	3+	cPR
13	+	40	3+	-	0	0	-	0	0	pCR
14	+	30	3+	-	0	0	-	+	0	pCR
15	+	0	0	-	0	0	+	0	0	cPR
16	+	20	2+	-	0	0	-	+	0	pCR
17	+	0	0	+	0	0	+	0	0	cSD
18	+	0	0	+	0	0	+	0	0	cPR

Abbreviations: +, present; -, absent; cPR, clinical partial response; pCR, pathologic complete response; cSD, clinical stable disease.

**Table 3:** CD44v status at 6 week biopsy and surgery stratified by response

CD44v at 6 week and surgery	pCR (N=8)	Non-pCR (N=10)	P
CD44v-	8 (100%)	4 (40%)	0.0128
CD44v +	0 (0%)	6 (60%)	0.0128

Abbreviations: +, present; -, absent; pCR, pathologic complete response.

## DISCUSSION

Our findings indicate that persistent or increased expression of CD44v positive cancer cells, or an increase in the expression level of CD44v after 6 weeks of lapatinib and trastuzumab therapy may predict unsuccessful induction of pCR in breast cancer patients treated with preoperative dual anti-HER2 therapy and concurrent cytotoxic chemotherapy. Biopsy during the course of preoperative treatment may provide information useful for guiding the application of therapeutic strategies that target CSCs.

Increased number of cells expressing CD44+/CD24- markers on treatment predicts resistance to cytotoxic therapy. However, concurrent treatment with lapatinib has been shown to dampen this increase [23], indicating that anti-HER2 therapy may provide a therapeutic strategy for eliminating resistant cells and decreasing recurrence rates. Persistent expression of CD44v or enrichment of CD44v during dual anti-HER2 therapy may be predictive of poor response to anti-HER2 therapy and concurrent cytotoxic chemotherapy in the preoperative setting. We examined various CSCs-related biomarkers and major signaling pathways regulating proliferation and survival of cancer cells that might predict pCR to preoperative dual anti-HER2 therapy. In our study, all patients who achieved pCR had a reduction in or disappearance of CD44v positive cells between baseline and 6 weeks or had no CD44v positive cells at either baseline or 6 weeks. Of the biomarkers for CSCs evaluated, CD44v was the only marker to show a statistically significant association with pCR. EMT markers in CTCs and the ratios of pEGFR/EGFR, pHER2/HER2, pERK/ERK, and pAkt/Akt in tumor tissue were not associated with pathologic response.

The pCR rate of 44% in our study was similar to that reported in the NeoALTTO study with preoperative combined anti-HER2 therapy. In that trial, preoperative therapy with the combination of trastuzumab and lapatinib resulted in a higher pCR rate than trastuzumab or lapatinib alone (51.5% vs 29.5%; *P* = 0.0001) [4, 5].

In a prior study, CD44+/CD24- expression was found to be a possible predictor of response to therapy with trastuzumab in patients treated with adjuvant therapy [22]. Similarly, our study found that CD44v persistence may be used as a prediction marker of response to therapy in patients with HER2+ breast cancer treated preoperatively with dual anti-HER2 therapy and concurrent cytotoxic chemotherapy, especially with agents that may target CSCs. However, our study was statistically designed to determine biomarkers changes, with not enough power for pCR detection, so we present the results of this clinical endpoint as a descriptive exploratory analysis. This finding has to be assessed in future trials with the clinical response as the primary endpoint. We are able to observe changes in the proportion of CD44v positive tumor cells as well as the intensity of the staining in the 6 week biopsy after the exposure to lapatinib and trastuzumab as well as the surgical specimen, after the exposure to lapatinib, trastuzumab and concurrent cytotoxic therapy. This result suggests a possible value of evaluating tissue biopsy during the course of therapy in addition to the baseline biopsy at the time of diagnosis, unlike other studies evaluating changes in a biopsy after the exposure to the whole treatment, we evaluated biomarker changes both after the exposure to dual anti-HER2 therapy alone and dual anti-HER2 therapy combined with chemotherapy. However, the optimal timing of tissue biopsy and the strategy during preoperative therapy cannot be answered from the current study. Although potential limitations of the study include the small number of patients and the statistical design to detect biomarker changes, our finding has suggested the potential importance of analysis of CSCs biomarker to predict therapy response and additional clinical studies are warranted for further exploration. Moreover, the prognostic role of CD44v persistence needs to be addressed in future trials with a greater accrual of patients. Currently, our choice of dual anti-HER2 therapy (trastuzumab/lapatinib) is not considered a standard preoperative therapy, but the pCR rate we achieved was substantial, and combination with lapatinib has been shown to potentially reduce the number of CD44v-expressing breast cancer cells [23]. Further, we believe that there is likelihood for the currently used dual anti-HER2 therapeutic strategy with trastuzumab and pertuzumab to result in the same outcome, but this should be confirmed in a future clinical trial.

In conclusion, we found that persistent expression of CD44v or enrichment of CD44v through preoperative therapy after dual anti-HER2 therapy may be predictive of poor response to dual anti-HER2 therapy with cytotoxic chemotherapy and may play a significant role in determining a successful therapeutic strategy for drugs that may target CSCs. Based on our findings, a single evaluation of biomarkers before therapy is insufficient for prediction of pathologic response.

Therefore, applying the adaptive response biopsy during the course of preoperative therapy may play a significant role in the success of therapeutic strategies that target CSCs.

## MATERIALS AND METHODS

### Participants

Eligible patients were Japanese women 20 years of age or older who had histologically or cytologically confirmed and previously untreated HER2+ invasive breast cancer (≥T2 excluding inflammatory breast cancer, any N, M0). HER2 positivity was defined following the published guideline by ASCO/CAP in 2007 [30], as a HER2 staining score of 3+ on immunohistochemistry (IHC) or a HER2 staining score of 2+ on IHC and a HER2/CEP17 ratio greater than 2.2 on fluorescence in situ hybridization. IHC was performed using PATHWAY anti-HER2/neu (4B5) rabbit monoclonal primary antibody (Ventana Medical Systems, Inc., Tucson, AZ). Patients were required to have an ECOG performance status of 0 or 1; adequate hematologic, renal, and hepatic function; left ventricular ejection fraction 50% or greater determined by multi-gated acquisition or echocardiography; and ability to take oral medications. We excluded patients who had received chemotherapy or radiotherapy or had a current uncontrolled illness, including an illness that would interfere with drug absorption (e.g., uncontrolled vomiting, inability to swallow, or chronic malabsorption).

### Study design

This was a single arm, open-label, phase II study (NCT01688609) to investigate putative biomarkers of CSCs and CTCs with EMT phenotype, alterations in quantities and phenotypes of CSCs and CTCs following anti-HER2 plus cytotoxic chemotherapy for primary operable HER2+ breast cancer in Japanese women. Eighteen patients were enrolled at Keio University Hospital (Tokyo, Japan) and St. Luke's International Hospital (Tokyo, Japan) from November 12, 2012, through July 2, 2014. The study and informed consent received Institutional Review Board (IRB) approval from St. Luke's international Hospital and Keio University Hospital.

### Treatment and procedures

All participants were required to undergo core needle biopsy for diagnosis as standard of care. Additional samples were collected via core needle biopsy for assay of putative biomarkers at baseline. During the first 6 weeks of the study, patients were given trastuzumab and lapatinib alone. Lapatinib was given at 1000 mg/day; trastuzumab was started with a loading dose of 4 mg/kg and was then given at a dose of 2 mg/kg weekly. Patients were also required to undergo a second core needle biopsy after the first 6-week study drug exposure with anti-Her2 therapy for a second biomarker assay. During the next 12 weeks, patients were given lapatinib at 750 mg daily and trastuzumab at 2 mg/kg weekly concurrently with paclitaxel at 80 mg/m^2^ weekly. At the end of these 12 weeks, patients underwent surgery, followed by postoperative treatment at the treating physician's discretion (4 cycles of fluorouracil 500 mg/m^2^, epirubicin 100 mg/m^2^, and cyclophosphamide 500 mg/m^2^, followed by 34 weeks of trastuzumab, was considered to be the standard adjuvant treatment) (Supplementary Figure 1).

To meet the primary objectives of the trial we i) determined the changes in the proportions of CD44v-positive (CD44v+) tumor cells and ALDH1-positive (ALDH1+) tumor cells in tumor tissue, EMT markers (TWIST1, SNAIL1, SLUG, ZEB1, and FOXC2) in CTCs in peripheral blood and the ratios of phosphorylated to phosphorylated EGFR (pEGFR/EGFR), HER2 (pHER2/HER2), ERK (pERK/ERK), and Akt (pAkt/Akt) in tumor tissues between baseline and at 6 weeks after initiation of preoperative therapy with anti-Her2 therapy, and between baseline and the end of concurrent trastuzumab, lapatinib, and paclitaxel therapy, ii) determined the pCR rate (defined as ypT0 ypN0) produced by lapatinib and trastuzumab followed by concurrent lapatinib, trastuzumab, and paclitaxel in patients with operable HER2+ breast cancer and iii) we assessed the safety and tolerability of study therapy in Japanese women using the Common Terminology Criteria for Adverse Events, version 4.0.

Response rate [complete response (CR) and partial response (PR)] was determined per Response Evaluation Criteria in Solid Tumors (RECIST) criteria 1.0 [31]. Axillary lymph node status was evaluated by ultrasound-guided fine needle aspiration for clinically positive nodes before the 6-week study drug exposure and by sentinel lymph node biopsy for patients with clinically negative nodes before the 6-week study drug exposure or after preoperative therapy. If the sentinel lymph node biopsy or fine needle aspiration was positive for metastasis, the patient underwent axillary lymph node dissection after the preoperative therapy. All patients were evaluated after completion of the concurrent preoperative trastuzumab, lapatinib, and paclitaxel. Patients had either lumpectomy or mastectomy and tissue was collected for evaluation of response to study therapy and for assay of research biomarkers.

### Special studies and technical details

Laboratory correlates were studied in 3 tumor tissue samples: (1) initial biopsy sample; (2) biopsy sample obtained after study drug exposure (at 6 weeks); and (3) sample collected during surgery. Blood samples were collected for CTC determination at the 3 points described above. The following biomarkers were tested at each time point: CD44v, ALDH1, EMT markers, pEGFR/EGFR, pHER2/HER2, pERK/ERK, pAkt/Akt, number of CTCs. Core needle biopsy samples obtained at diagnosis were examined by routine histopathology evaluation with hematoxylin and eosin staining and with IHC staining for hormone receptors and HER2-expression status, CD44v, and ALDH1.

HER2 expression status was determined by IHC and fluorescence in situ hybridization. IHC staining was performed at local laboratories using a rabbit monoclonal antibody (Ventana clone 4B5) and following the published guideline by ASCO/CAP in 2007 [30]. On IHC staining, a score of 3+ was defined as HER2+, and a score of 0 or 1+ was defined as HER2 negative. When the score on IHC was 2+, fluorescence in situ hybridization was used to determine HER2 status. In such cases, when the HER2/CEP17 ratio was greater than 2.2, the patient was considered to have HER2+ breast cancer.

For CD44v staining, samples were incubated with anti-CD44v antibodies (1:1000, rat monoclonal; described by Ishimoto et al., [9]). Primary antibodies were then visualized by incubating cells with Alexa 488-conjugated goat anti-mouse antibodies (1:1000; Invitrogen, ThermoFisher Scientific, Waltham, MA) and Alexa 594-conjugated goat anti-rat antibodies (1:1000; Invitrogen) for 1 hour at 37°C. After incubation with the secondary antibodies, sections were rinsed 3 times with phosphate-buffered saline. The samples were examined by fluorescence microscopy (Zeiss, Tokyo, Japan). CD44v+ tumor cells were identified using the imaging analysis software Axiovision, version 4.8 (Zeiss). Only carcinoma foci were selected based on the hematoxylin and eosin staining. Protein expression was evaluated by fluorescence level intensity (gray scale). This method identified 2 tumor cell types that are distinguished by their CD44v expression pattern, CD44v+ and CD44v-negative. According to these standard criteria, the ratio of CD44v+ tumor cells to the total number of counted carcinoma cells was expressed as percentage of CD44v+ cells [32] (Figure 2). ALDH1 antibodies (abcam catalog #ab52492; rabbit monoclonal antibody, clone EP1933Y, BD Biosciences, San Jose, CA) were used to detect ALDH1. Expression of ALDH1 was measured by IHC. A sample with at least 1 ALDH1+ cancer cell was considered ALDH1+.

EMT markers (TWIST1, SNAIL1, SLUG, ZEB1, and FOXC2) in CTCs in peripheral blood were measured by quantitative reverse transcription polymerase chain reaction. CTCs were enumerated by CellSearch (Janssen Diagnostics, South Raritan, NJ) and AdnaTest (AdnaGen, San Antonio, TX) [33] (Supplementary Table 1). The ratios of pEGFR/EGFR, pHER2/HER2, pERK/ERK, and pAkt/Akt in tumor tissues were measured by laser scanning cytometry using ApoCell (ApoCell, Inc., Houston, TX) technology as performed by Davis [29].

### Statistical analysis

This study was designed to meet the primary end point (biomarker change) with the clinical endpoint (pCR) as an exploratory analysis. Associations between categorical variables and pCR vs non-pCR were assessed via cross-tabulation and Fisher's exact test. Changes in the binary biomarkers between time points were assessed using McNemar's test in all patients and separately in patients with and without pCR. Changes in the continuous biomarkers between time points were estimated and compared between the pCR and non-pCR groups using Wilcoxon rank-sum test.

## SUPPLEMENTARY MATERIALS FIGURES AND TABLES



## Abbreviations

5-FU: 5-Flourouracil
ALDH1: Aldehyde dehydrogenase 1
ALT: Alanine aminotransferase
CR: Complete response
CSC's: Cancer stem cells
cSD: Clinical stable disease
CTCAE: Common terminology criteria for adverse events
CTS's: Circulating tumor cells
Ctx: Cyclophosphamide
ECOG: Eastern cooperative oncology group scale of performance status
EGFR: Epidermal growth factor receptor
EMT: Epithelial-Mesenchymal Transition
Epi: Epirubicin
ER: Estrogen receptor
GGT: Gamma-glutamyl transpeptidase
HER2: Human epidermal growth factor receptor 2
IHC: Immunohistochemistry
pCR: Pathologic complete response
PR: Partial response
PR: Progesterone receptor
RECIST: Response evaluation criteria in solid tumors
VEGFR: Vascular endothelial growth factor receptor

## References

[1] Mauri D, Pavlidis N, Ioannidis JP Neoadjuvant versus adjuvant systemic treatment in breast cancer: a meta-analysis. J Natl Inst.

[2] Gianni L, Eiermann W, Semiglazov V, Manikhas A, Lluch A, Tjulandin S, Zambetti M, Vazquez F, Byakhow M, Lichinitser M, Climent MA, Ciruelos E, Ojeda B Neoadjuvant chemotherapy with trastuzumab followed by adjuvant trastuzumab versus neoadjuvant chemotherapy alone, in patients with HER2-positive locally advanced breast cancer (the NOAH trial): a randomised controlled superiority trial with a parallel HER2-negative cohort. Lancet.

[3] Untch M, Fasching PA, Konecny GE, Hasmuller S, Lebeau A, Kreienberg R, Camara O, Müller V, du Bois A, Kühn T, Stickeler E, Harbeck N, Höss C Pathologic complete response after neoadjuvant chemotherapy plus trastuzumab predicts favorable survival in human epidermal growth factor receptor 2-overexpressing breast cancer: results from the TECHNO trial of the AGO and GBG study groups. J Clin Oncol.

[4] Baselga J, Bradbury I, Eidtmann H, Di Cosimo S, de Azambuja E, Aura C, Gómez H, Dinh P, Fauria K, Van Dooren V, Aktan G, Goldhirsch A, Chang TW Lapatinib with trastuzumab for HER2-positive early breast cancer (NeoALTTO): a randomised, open-label, multicentre, phase 3 trial. Lancet.

[5] de Azambuja E, Holmes AP, Piccart-Gebhart M, Holmes E, Di Cosimo S, Swaby RF, Untch M, Jackisch C, Lang I, Smith I, Boyle F, Xu B, Barrios C Lapatinib with trastuzumab for HER2-positive early breast cancer (NeoALTTO): survival outcomes of a randomised, open-label, multicentre, phase 3 trial and their association with pathological complete response. Lancet Oncol.

[6] Sugihara E, Saya H Complexity of cancer stem cells. Int J Cancer.

[7] Nguyen NP, Almeida FS, Chi A, Nguyen LM, Cohen D, Karlsson U, Vinh-Hung V Molecular biology of breast cancer stem cells: potential clinical applications. Cancer Treat Rev.

[8] Nagano O, Saya H Mechanism and biological significance of CD44 cleavage. Cancer Sci.

[9] Ishimoto T, Nagano O, Yae T, Tamada M, Motohara T, Oshima H, Oshima M, Ikeda T, Asaba R, Yagi H, Masuko T, Shimizu T, Ishikawa T CD44 variant regulates redox status in cancer cells by stabilizing the xCT subunit of system xc− and thereby promotes tumor growth. Cancer Cell.

[10] Yoshikawa M, Tsuchihashi K, Ishimoto T, Yae T, Motohara T, Sugihara E, Onishi N, Masuko T, Yoshizawa K, Kawashiri S, Mukai M, Asoda S, Kawana H xCT inhibition depletes CD44v-expressing tumor cells that are resistant to EGFR-targeted therapy in head and neck squamous cell carcinoma. Cancer Res.

[11] Yae T, Tsuchihashi K, Ishimoto T, Motohara T, Yoshikawa M, Yoshida GJ, Wada T, Masuko T, Mogushi K, Tanaka H, Osawa T, Kanki Y, Minami T Alternative splicing of CD44 mRNA by ESRP1 enhances lung colonization of metastatic cancer cell. Nat Commun.

[12] Nagano O, Okazaki S, Saya H Redox regulation in stem-like cancer cells by CD44 variant isoforms. Oncogene.

[13] Ginestier C, Hur MH, Charafe-Jauffret E, Monville F, Dutcher J, Brown M, Jacquemier J, Viens P, Kleer CG, Liu S, Schott A, Hayes D, Birnbaum D ALDH1 is a marker of normal and malignant human mammary stem cells and a predictor of poor clinical outcome. Cell Stem Cell.

[14] Cristofanilli M, Hayes DF, Budd GT, Ellis MJ, Stopeck A, Reuben JM, Doyle GV, Matera J, Allard WJ, Miller MC, Fritsche HA, Hortobagyi GN, Terstappen LW Circulating tumor cells: a novel prognostic factor for newly diagnosed metastatic breast cancer. J Clin Oncol.

[15] Dawood S, Broglio K, Valero V, Reuben J, Handy B, Islam R, Jackson S, Hortobagyi GN, Fritsche H, Cristofanilli M Circulating tumor cells in metastatic breast cancer: from prognostic stratification to modification of the staging system?. Cancer.

[16] Mani SA, Guo W, Liao MJ, Eaton EN, Ayyanan A, Zhou AY, Brooks M, Reinhard F, Zhang CC, Shipitsin M, Campbell LL, Polyak K, Brisken C The epithelial-mesenchymal transition generates cells with properties of stem cells. Cell.

[17] Sato R, Semba T, Saya H, Arima Y Concise review: stem cells and epithelial-mesenchymal transition in cancer: biological implications and therapeutic targets. Stem Cells.

[18] Liu H, Lv L, Yang K Chemotherapy targeting cancer stem cells. Am J Cancer Res.

[19] Martin-Castillo B, Lopez-Bonet E, Cuyàs E, Viñas G, Pernas S, Dorca J, Menendez JA Cancer stem cell-driven efficacy of trastuzumab (Herceptin): towards a reclassification of clinically HER2-positive breast carcinomas. Oncotarget.

[20] Li HZ, Yi TB, Wu ZY Suspension culture combined with chemotherapeutic agents for sorting of breast cancer stem cells. BMC Cancer.

[21] Tanei T, Morimoto K, Shimazu K, Kim SJ, Tanji Y, Taguchi T, Tamaki Y, Noguchi S Association of breast cancer stem cells identified by aldehyde dehydrogenase 1 expression with resistance to sequential Paclitaxel and epirubicin-based chemotherapy for breast cancers. Clin Cancer Res.

[22] Seo AN, Lee HJ, Kim EJ, Jang MH, Kim YJ, Kim JH, Kim SW, Ryu HS, Park IA, Im SA, Gong G, Jung KH, Kim HJ, Park SY Expression of breast cancer stem cell markers as predictors of prognosis and response to trastuzumab in HER2-positive breast cancer. Br J Cancer.

[23] Li X, Lewis MT, Huang J, Gutierrez C, Osborne CK, Wu MF, Hilsenbeck SG, Pavlick A, Zhang X, Chamness GC, Wong H, Rosen J, Chang JC Intrinsic resistance of tumorigenic breast cancer cells to chemotherapy. J Natl Cancer Inst.

[24] Magnifico A, Albano L, Campaner S, Delia D, Castiglioni F, Gasparini P, Sozzi G, Fontanella E, Menard S, Tagliabue E Tumor-initiating cells of HER2-positive carcinoma cell lines express the highest oncoprotein levels and are sensitive to trastuzumab. Clin Cancer Res.

[25] Paik S, Kim C, Wolmark N HER2 status and benefit from adjuvant trastuzumab in breast cancer. N Engl J Med.

[26] Aktas B, Tewes M, Fehm T, Hauch S, Kimmig R, Kasimir-Bauer S Stem cell and epithelial-mesenchymal transition markers are frequently overexpressed in circulating tumor cells of metastatic breast cancer patients. Breast Cancer Res.

[27] Dowlati A, Haaga J, Remick SC, Spiro TP, Gerson SL, Liu L, Berger SJ, Berger NA, Willson JK Sequential tumor biopsies in early phase clinical trials of anticancer agents for pharmacodynamic evaluation. Clin Cancer Res.

[28] Ang JE, Kaye S, Banerji U Tissue-based approaches to study pharmacodynamic endpoints in early phase oncology clinical trials. Curr Drug Targets.

[29] Cohen EE, Davis DW, Karrison TG, Seiwert TY, Wong SJ, Nattam S, Kozloff MF, Clark JI, Yan DH, Liu W, Pierce C, Dancey JE, Stenson K Erlotinib and bevacizumab in patients with recurrent or metastatic squamous-cell carcinoma of the head and neck: a phase I/II study. Lancet Oncol.

[30] Wolff AC, Hammond ME, Schwartz JN, Hagerty KL, Allred DC, Cote RJ, Dowsett M, Fitzgibbons PL, Hanna WM, Langer A, McShane LM, Paik S, Pegram MD American Society of Clinical Oncology/College of American Pathologists guideline recommendations for human epidermal growth factor receptor 2 testing in breast cancer. J Clin Oncol.

[31] Therasse P, Arbuck SG, Eisenhauer EA, Wanders J, Kaplan RS, Rubinstein L, Verweij J, Van Glabbeke M, van Oosterom AT, Christian MC, Gwyther SG New guidelines to evaluate the response to treatment in solid tumors. European Organization for Research and Treatment of Cancer, National Cancer Institute of the United States, National Cancer Institute of Canada. J Natl Cancer Inst.

[32] Kai M, Onishi H, Souzaki M, Tanaka H, Kubo M, Tanaka M, Katano M Semi-quantitative evaluation of CD44+/CD24− tumor cell distribution in breast cancer tissue using a newly developed fluorescence immunohistochemical staining method. Cancer Sci.

[33] Mani SA, Yang J, Brooks M, Schwaninger G, Zhou A, Miura N, Kutok JL, Hartwell K, Richardson AL, Weinberg RA Mesenchyme Forkhead 1 (FOXC2) plays a key role in metastasis and is associated with aggressive basal-like breast cancers. Proc Natl Acad Sci U S A.

