# Supplementary material for: Dynamic changes in CD44v-positive cells after preoperative anti-HER2 therapy and its correlation with pathologic complete response in HER2-positive breast cancer
Source: Oncotarget. 2018 Jan 4;9(6):6872–82. doi: 10.18632/oncotarget.23914 (PMC5805522; doi:10.18632/oncotarget.23914)
Supplement: Supplementary file 1 [file oncotarget-09-6872-s001.pdf]

Dynamic changes in CD44v-positive cells after preoperative anti-HER2 therapy and its correlation with pathologic complete response in HER2-positive breast cancer

SUPPLEMENTARY MATERIALS

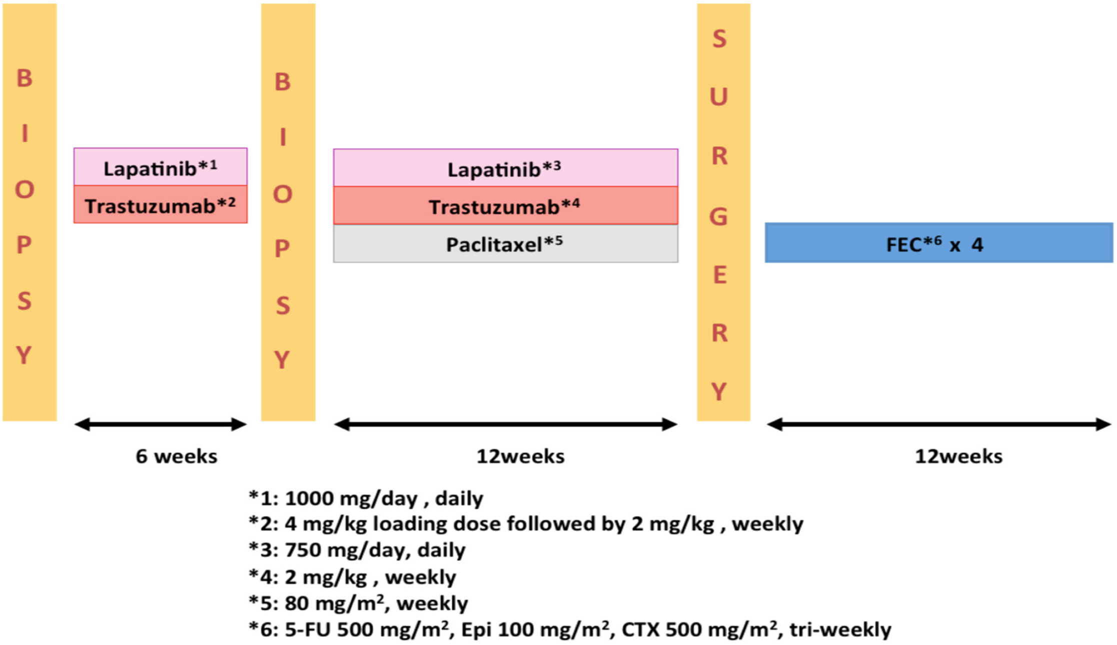

Supplementary Figure 1: Trial profile. Abbreviations: 5-FU, 5-Flourouracil; Epi, Epirubicin; Ctx, Cyclophosphamide.

Supplementary Table 1: CTC detection by CellSearch and AdnaTest

Patient	Before Study		End of Week 6		Before Surgery	
	AdnaTest	CellSearch	AdnaTest	CellSearch	AdnaTest	CellSearch
1	0	0	0	0	0	0
2	0	1	0	6	0	0
3	EpCAM	1	0	0	+	0
4	0	2	0	2	0	0
5	0	2	0	0	0	1
6	EpCAM/ MUC1	5	MUC1	3	MUC1	0
7	0	0	0	1	0	0
8	0	0	0	1	0	2
9	0	0	0	0	0	N/A
10	0	0	0	0	0	0
11	0	0	0	0	0	1
12	HER2/MUC1	1	0	0	HER2/MUC1	2
13	MUC1	0	MUC1/EpCAM	0	MUC1	0
14	0	0	0	N/A	0	0
15	0	0	MUC1	0	0	1
16	0	1	0	0	0	1
17	0	1	0	0	MUC1	6
18	0	3	0	3	0	3

Abbreviations: CTC, circulating tumor cells; +, present; 0, absent; N/A, not available AdnaTest considered positive by the presence of any marker; Her2, EpCAM, MUC1 CellSearch expressed as the number of CTCs detected.

Supplementary Table 2: Biomarkers and pCR prediction

Marker	Base Line			End fo Week 6			Before Surgery		P
	pCR (n)	Non-pCR (n)	P	pCR (n)	Non-pCR (n)	P	pCR (n)	Non- pCR (n)	
ALDH1	8	10	0.1017	7	10	0.1651	8	9	1.0000
EMT/CTC	4	8	0.3213	4	8	0.5637	4	8	0.1797
pHER2/HER2 ratio	8	9	0.1553	2	8	0.7044	3	8	0.4913
pEGFR/EGFR ratio	8	9	0.9622	2	8	0.8990	3	8	0.7657
pERK/ERK ratio	8	9	0.6708	2	8	0.8990	3	8	0.2878
pAKT/AKT ratio	7	9	1.0000	1	8	0.3613	2	8	0.5302

Abbreviations: EMT-CTC presence of any gene transcript (EpCAM, FOXc2, KRT19, SNAIL1, SNAIL2, TWIST1, ZEB1); pCR, pathologic complete response
